# Health Tract, No. 30

**Published:** 1865

**Authors:** 


### HEALTH TRACT, No. 30.

### BATHS AND BATHING.

PIGS, puppies, and babies are the better for being well washed every day, but for persons in general to undergo such an operation as regular as the morning comes is absurd and hurtful. Absurd, because unnecessary, and no man ever did it for a lifetime; hurtful, because multitudes who commenced the unnatural practice, have abandoned it from the conviction that it had an unfavorable effect, or that they ceased to be benefited by it.

It is proper that once a week there should be a most thorough washing of the whole body with soap, water at about eighty degrees Fahrenheit, and a common scrubbing-brush. To avoid taking cold, especially in winter, the heat of the room should be within six or eight degrees of that of the water.

The whole operation, from the time of beginning to undress until completed, should not exceed twenty minutes, including the friction, which should be rapid and thorough, with a coarse towel.

Microscopists say that the skin of a man is like the scales of a fish, which are covered with a slimy substance, to throw off the water and also to lubricate the scales so that they may slide over each other with the greatest facility. If this lubricator were kept washed from the fish, it would die. It may be inferred that the oil which nature throws out on the skin is designed for the wise purpose of a lubricator, to keep the skin moist and soft and smooth. In severe fever or cold, the dry harsh skin, and the "goose flesh," are familiar to all; in both of which there is an entire absence of perspiration, and relief comes only with perspiration. Let all think for themselves in this matter.

Much is said about the universality of bathing among the Romans. The practice did not become general until national voluptuousness, gormandizing and intemperance were destroying the national vigor; but their magnificent bathing establishments, public and private, failed to restore individual health or to prevent national ruin. We are told that the "Eastern Nations" practice bathing. Suppose they do; they are the filthiest people on the face of the globe, as to the Moors, Turks, Hindoos, East-Indians, Chinese, Japanese, etc., while the average of human life is less than our own by many years; and their great men and great deeds and magnificent achievements, where are they?

The masses with us have imperative duties to perform, and can not afford to spend an hour every day in wriggling and splashing and spluttering about in cold water; and happily health does not require it, either of the day laborer or of the man of elegant leisure; all that is needed for either, beyond the weekly bath named, is to wash the exposed parts morning, and in some cases, evening too, most thoroughly; that is, the hands, face, neck, throat, arms, and armpits. Beyond this is not indicated either by common-sense or a rational physiology.

